# Comprehension of the adapted Urticaria Activity Score measure and patient guidance document: qualitative interviews with adults and adolescents with chronic spontaneous urticaria

**DOI:** 10.1186/s41687-024-00830-9

**Published:** 2024-12-24

**Authors:** Anne M. Skalicky, Yan Wang, Olabimpe R. Eseyin, Marissa Stefan, Pallavi B. Rane, Julie McLaren, Marcus Maurer

**Affiliations:** 1 Evidera, Inc., 929 North Front Street, Wilmington, NC 28401 USA; 2 Amgen Inc., One Amgen Center Drive, Thousand Oaks, CA USA; 3 Institute of Allergology, Charité – Universitätsmedizin Berlin, Corporate Member of Freie Universität Berlin and Humboldt-Universität zu Berlin, Paul-Ehrlich-Haus, Hindenburgdamm 30, 12203 Berlin, Germany; 4 Fraunhofer Institute for Translational Medicine and Pharmacology ITMP, Allergology and Immunology, Berlin, Germany

**Keywords:** Qualitative interviews, Content validation, Chronic spontaneous urticaria, Urticaria Activity Score, Adults, Adolescents

## Abstract

**Objective:**

A key component of determining that a patient-reported outcome (PRO) measure is fit-for-purpose is to ensure that respondents understand its instructions and items. Any modification to a measure should be evaluated for relevance and understandability. The objective of the study was to assess comprehension of the adapted Urticaria Activity Score (UAS) questionnaire among adolescents aged populations with chronic spontaneous urticaria (CSU) and the modification to UAS question 2 to include patient-friendly terminology “wheals (hives).” A patient guidance document for completing the adapted UAS was also examined.

**Methods:**

A non-interventional, cross-sectional, qualitative study involving hybrid concept elicitation and cognitive interviews was conducted among adults and adolescents with CSU. Eligibility included clinician confirmation of CSU and experience of itch and hives for ≥ 6 weeks. Study participants were recruited from US clinical sites and online CSU patient communities. Telephone interviews were conducted using a semi-structured interview guide. Participants reviewed the UAS and provided their input on the UAS guidance document.

**Results:**

Twenty-two interviews were conducted (seven adolescents and 15 adults; mean age 34 ± 18 years, 64% female, 77% White, 77% non-Hispanic, and 59% moderate to severe CSU symptoms). All participants (*n* = 22/22, 100%) stated that the adapted UAS was clear and “easy” to understand. For the adapted UAS “wheals (hives)” item, several participants were unfamiliar with the term “wheals,” but the term “hives” was well understood by US participants. Most participants reported that it was “easy” or “not difficult” to count and recall the number of hives they had over the past 24 h. Participants found the adapted UAS guidance document “helpful” and “easy to understand” for determining and counting hives in a 24-hour period. Suggestions for improving the guide included adding a picture to aid in counting hives.

**Conclusions:**

Itch and hives are important symptoms of CSU. Results support the content validity of an adapted UAS as a daily measure of severity of itch and hives and provided valuable suggestions for improving its patient guidance, which can be used in future clinical trials involving adults and adolescents ≥ 12 years old with CSU to assess the severity of itch and hives.

**Supplementary Information:**

The online version contains supplementary material available at 10.1186/s41687-024-00830-9.

## Background

Chronic spontaneous urticaria (CSU) is characterized by the occurrence of itchy wheals/hives, angioedema, or both for 6 weeks or more [1–4]. There are no clear causes of CSU. People living with CSU can experience fluctuating severity of itch and hives.

The original Urticaria Activity Score (UAS) is a commonly used diary-based patient-reported outcome (PRO) measure that assesses the key sign (wheals) and symptom (itch) of CSU [4]. The first question asks respondents how intense their itch has been over the past 24 h, and the second asks respondents to report the number of wheals they had over the past 24 h. Specific terms such as “wheals” may not be used commonly in the US. Daily UAS scores are summed over 7 consecutive days to create the weekly UAS (UAS7) score, and higher scores indicate greater disease severity.

No publications are available to confirm the validity of the UAS in adolescents. To ensure the use of patient-friendly terminology, the original UAS was adapted (with the developer’s permission) to explain the lesser-known term “wheals” in the US version. Additionally, a patient guidance document was developed with specific instructions to aid completion of the adapted UAS and minimize variability in individual responses.

The purpose of this study was to assess the content validity of the adapted UAS for use in treatment trials with adolescent and adult patients with CSU through cognitive interviews and to assess the understanding of the patient guidance document for completing the adapted UAS. To address the US FDA’s recommendation to include patient-friendly terminology in clinical outcome assessments, we conducted cognitive interviews with individuals from the target patient population to guide the choice of patient-friendly terminology (e.g., “hives” and “itch” rather than “wheals” and “pruritus,” respectively) to use in the PRO measure to maximize the comprehensibility of the instrument across individuals with different levels of health literacy.

## Method

### Study design

This was a non-interventional, cross-sectional, qualitative study involving three iterative rounds of semi-structured, hybrid concept elicitation/cognitive interviews. Each round included interviews with different participants who discussed their CSU symptoms, reviewed the adapted UAS—including the revised terminology of “wheals (hives)”—and discussed their comprehension of the patient guidance document for completing the adapted UAS. The aim of conducting cognitive interviews in rounds is to assess, between rounds, whether changes to the UAS would be needed. The instrument review process for the adapted UAS was guided by procedures outlined by the Professional Society for Health Economics and Outcomes Research (ISPOR) PRO Good Practice Task Force, Part II [5] and in accordance with the FDA’s Patient-Focused Drug Development guidance [6].

### Patient population and recruitment

The study population, diverse with respect to age, gender, race, ethnicity, health literacy, and clinical severity, consisted of adolescents 12 to 17 years old and adults 18 to 80 years old who had moderate to severe CSU. Individuals with CSU were eligible if they had self-reported moderate to severe itch and hives for ≥ 6 weeks (adults) or a physician-reported diagnosis of moderate to severe CSU (adolescents), clinical confirmation of CSU, spontaneously occurring itch and hives for ≥ 6 consecutive weeks, itch and hive symptoms occurring on most days of the week (e.g., 4 out of 7 days), and uncontrolled symptoms despite treatment with a second-generation antihistamine (up to four times the approved dose). Individuals were ineligible if they had a history of skin disease or the presence of a skin condition—other than those related to CSU—that would interfere with describing their experience living with CSU (e.g., atopic dermatitis, bullous pemphigoid, dermatitis herpetiformis, senile pruritus).

Adult participants were identified through a research recruitment vendor using multiple sources (e.g., patient databases, physician referrals, social media, patient associations, and support groups). Adolescent participants were identified through three pediatric clinical sites and the research recruitment vendor.

This study was conducted in accordance with the ethical principles based on the Declaration of Helsinki and was approved by an institutional review board (Advarra CIRBITM; Pro00065619). All participants provided informed consent electronically.

Before study enrollment, eligibility was ascertained through a screening procedure, and verification of CSU diagnosis was confirmed by photographed or scanned medical records. Once diagnosis was confirmed, adult participants were consented, and adolescent participants and their parent/caregiver provided assent and consent, respectively. Enrolled participants were provided a sociodemographic and clinical characteristics questionnaire to complete before scheduling their interview.

### Study measures

#### Adapted UAS

The UAS is a two-item PRO that evaluates CSU disease activity. The first item asks individuals to indicate the intensity of their itch over the past 24 h (none, mild, moderate, intense), and the second item asks about number of wheals over the past 24 h (none, < 20, 20 to 50, > 50). The responses are scored from 0 (no wheals, no itch) to 3 (many wheals, intense itch) for each question. The summed score from the two questions gives a daily UAS score (0 to 6), and 7 consecutive days of scores gives a UAS7 (0 to 42) score; higher scores indicate greater disease severity. Permission to revise the UAS was obtained from the UAS copyright holder [7, 8]. The adapted UAS involved modifying the UAS wheals item to read “wheals (hives)” to improve patient comprehension for adults and adolescents who may not be familiar with the term “wheals” (see supplementary materials).

#### Patient guidance document

A two-page patient guidance document for completing the adapted UAS was developed. The patient guidance document was written at a seventh-grade reading level (assessed using the Flesch-Kincaid reading [9]) and provides guidance on how to assess itch and wheals (hives) to answer the adapted UAS. The patient guidance document was provided to ensure comprehension of the UAS (see supplementary materials).

### Interview process

All study participants were invited to complete a single, one-on-one interview via telephone or video conference. Participants were mailed a study packet before the interview that included the adapted UAS and the patient guidance document. All interviews were audio-recorded. At the start of the interview, participants were asked open-ended questions about their CSU symptoms. After the concept-elicitation portion, they were instructed to open the sealed envelope and read the patient guidance document before completing the adapted UAS. The cognitive interviews focused on assessing the participants’ comprehension of the UAS and its relevance, and the perceived meaningful change on the UAS item response scale based on hypothetical treatment change, and understanding/usefulness of the patient guidance. Participants were compensated for their time and effort.

### Data management and analysis

Demographic and clinical characteristic descriptive statistics were analyzed (using SAS statistical software, version 9.4) to characterize the sample. A content analysis approach was used to analyze the interview data, using ATLAS.ti (version 22; ATLAS.ti Scientific Software Development GmbH, Berlin, Germany). Codes were grouped by age and synthesized according to feedback on the adapted UAS and patient guidance document. Specific information was coded related to comprehension of the instructions, recall period, response scales, and meaningful change for the adapted UAS items. Saturation for CSU symptoms was assessed.

## Results

### Patient population

Study participants were interviewed between November 2022 and March 2023 in the US. Twenty-two participants (15 adults [68.2%] and seven adolescents [31.8%]) were interviewed. Adolescent participants were enrolled from three clinical sites in Arizona, Arkansas, and Indiana (*n* = 5, 22.7%); 77.3% were recruited via an online vendor (adults, *n* = 15 [100.0%]; adolescents, *n* = 2 [28.6%]). The mean age of the study population was 34 ± 17.9 years (adults, 43.3 ± 13.8 years [range: 12–17 years]; adolescents, 14.3 ± 1.8 years [range: 23–65 years]). Most of the adult sample was female (*n* = 11, 73.3%), whereas most of the adolescent sample was male (*n* = 4, 57.1%). Of the total sample, 77.3% were non-Hispanic and 77.3% were White. Participants were diagnosed on average 3 ± 3.0 years ago and had been receiving treatment for an average of 2.7 ± 2.3 years (adults, 2.8 ± 2.1 years; adolescents, 2.4 ± 2.6 years). Nearly two-thirds of the participants were from the southeastern (31.8%) or midwestern (31.8%) US. Most adolescent participants felt fairly confident (42.9%) or quite confident (28.6%) in their health literacy. Most adults felt extremely confident (86.7%) in their health literacy (Table 1).

**Table 1:** Demographic and clinical characteristics

Characteristic	Total (*N* = 22)	Adolescents (*n* = 7)	Adults (*n* = 15)
Age (years)			
N	22	7	15
Mean (SD)	34.0 (17.9)	14.3 (1.8)	43.3 (13.8)
Median (range)	33.5 (12–65)	14.0 (12–17)	40.0 (23–65)
Sex, n (%)			
Male	8 (36.4%)	4 (57.1%)	4 (26.7%)
Female	14 (63.6%)	3 (42.9%)	11 (73.3%)
Ethnic background, n (%)			
Hispanic or Latino	5 (22.7%)	1 (14.3%)	4 (26.7%)
Not Hispanic or Latino	17 (77.3%)	6 (85.7%)	11 (73.3%)
Racial background, n (%)			
Asian	1 (4.5%)	0 (0.0%)	1 (6.7%)
Black or African American	3 (13.6%)	2 (28.6%)	1 (6.7%)
White	17 (77.3%)	5 (71.4%)	12 (80.0%)
Other^a^	1 (4.5%)	0 (0.0%)	1 (6.7%)
Health literacy/confidence			
Extremely confident	14 (63.6%)	1 (14.3%)	13 (86.7%)
Quite confident	4 (18.2%)	2 (28.6%)	2 (13.3%)
Fairly confident	3 (13.6%)	3 (42.9%)	0 (0.0%)
A little confident	0 (0%)	0 (0%)	0 (0%)
Not confident	1 (4.5%)	1 (14.3%)	0 (0.0%)
Year diagnosed			
Mean (SD)	4 (3.2)	4 (3.2)	2 (2.3)
General health, n (%)			
Excellent	1 (4.5%)	1 (14.3%)	0 (0.0%)
Very good	9 (40.9%)	5 (71.4%)	4 (26.7%)
Good	8 (36.4%)	1 (14.3%)	7 (46.7%)
Fair	4 (18.2%)	0 (0.0%)	4 (26.7%)
Poor	0 (0%)	0 (0%)	0 (0%)
UAS Itch Item			
Mean (SD)	2.6 (1.0)	2.5 (0.9)	2.7 (1.1)
None	3 (13.6%)	2 (13.3%)	1 (14.3%)
Mild (present but not annoying or troublesome)	7 (31.8%)	5 (33.3%)	2 (28.6%)
Moderate (troublesome but does not interfere with normal daily activity or sleep)	8 (36.3%)	6 (40.0%)	2 (28.6%)
Intense (interferes with normal activity or sleep)	4 (18.2%)	2 (13.3%)	2 (28.6%)
UAS Wheals (hives) Item			
Mean (SD)	2.4 (0.9)	2.7 (1.1)	2.1 (0.7)
None	4 (18.1%)	1 (14.3%)	3 (20.0%)
<20	9 (40.9%)	2 (28.6%)	7 (46.7%)
20 to 50	7 (31.8%)	2 (28.6%)	5 (33.3%)
>50	2 (9.1%)	2 (28.6%)	0 (0%)

*SD* standard deviation, *UAS* Urticaria Activity Score

^a^Other race: Jewish

### Concept elicitation

Saturation of CSU concepts was reached within the first five interviews for itch, hives, angioedema, and swelling in the adult and adolescent groups. Most participants (total, *n* = 19/22, 86.4%; adults, *n* = 13/15, 86.7%; adolescents, *n* = 6/7, 85.7%) used the term “hives” when describing CSU. Only a few adults (*n* = 4/15, 26.7%) had heard of the term “wheals” or used it to describe their hives. Adult and adolescent participants reported that their CSU symptom severity varied from day to day and from flare-up to flare-up, with no clear, detectable pattern. Participants described how hives can differ in size and spread all over the body. Participants also noted that hives varied in the degree of itchiness and described nighttime itch as the most bothersome.

***100-119 Adult Male***: There are days that I have to control myself, and you can go out of your mind. There are days when it’s just milder and it’s more moderate. Sometimes it’s beyond moderate. Sometimes it’s before, a little under moderate. But on moderate days I can control it. On other days it’s very difficult to control and I rely on the medication….

***400-003 Adolescent Male***: During the night it keeps me up, and it gets worse. Like it spreads faster than what it would be like in the day.

***300-007 Adolescent Male***: It’s usually slightly flared up in the morning, and then I take the medicine, and then throughout most of the day, it’s pretty moderate, and then, whenever I get back home, at night, it starts to flare back up again.

Commonly suspected triggers included temperature/weather (total, *n* = 10/22, 45.5%; adults, *n* = 7/15, 46.7%; adolescents, *n* = 3/7, 42.9%), clothing (total, *n* = 8/22, 53.3%; adults, *n* = 7/15, 46.7%; adolescents, *n* = 1/7, 14.3%), stress (total, *n* = 8/22, 53.3%; adults, *n* = 6/15, 40.0%; adolescents, *n* = 2/7, 28.6%), food (total, *n* = 4/22, 18.2%; adults, *n* = 3/15, 20.0%; adolescents, *n* = 1/7, 14.3%), water (adults, *n* = 1/15, 6.7%), and environmental pollutants such as the chemicals in a swimming pool (total, *n* = 2/22, 9.1%; adults, *n* = 1/15, 6.7%; adolescents, *n* = 1/7, 14.3%).

### Feedback on adapted UAS

No changes were needed for the adapted UAS between interview rounds.

#### UAS item responses

Most adults reported mild (*n* = 5/15, 33.3%) or moderate (*n* = 6/15, 40.0%) itch severity and < 20 hives (*n* = 7/15, 46.7%) or 20 to 50 hives (*n* = 5/15, 33.3%) over the past 24 h. Adolescents reported having mild, moderate, or intense itch severity (*n* = 2/7, 28.6% each) and < 20, 20 to 50, or > 50 hives (*n* = 2/7, 28.6% each) over the past 24 h.

#### Clarity and relevance

All participants (*n* = 22/22, 100.0%) stated that the UAS was clear and “easy” to understand. All participants were able to describe the UAS items in their own words and demonstrated a clear understanding of the itch and wheals items. Most adults (*n* = 10/15, 66.7%) and all adolescents (*n* = 7/7, 100.0%) reported that the UAS recall period was easy to understand. Most of the participants reported that item phrasing—including the response choices—was clear for both items, and they were able to give accurate explanations of the symptom terms on which they had received in-depth instruction.

#### Understanding of the UAS itch intensity item

Participants were asked to explain what they understood or provide synonyms of the terms “score” and “intensity” in relation to itch. For adults, the synonyms provided for the term “score” included “choose” (*n* = 3/15, 20.0%); “rate,” “pick,” or “select” (*n* = 2/15, 13.3% each); “best describes,” “identify,” or “assess worst” (*n* = 1/15, 6.7% each). For adolescents, the synonyms provided for word “score” included “choose” (*n* = 3/7, 42.9%) and “select” (*n* = 2/7, 28.6%).

The term “intensity” was understood by adults as “how itchy” or “itchiness” (*n* = 3/15, 20.0% each), “level” of itch (*n* = 3/15, 20.0%), “severity” (*n* = 3/15, 20.0%), and “strength” or “impact” (*n* = 1/15, 6.7% each) of itch. Other synonyms for “intensity” used by adults included “how bad,” “annoying,” or “disruptive” and “how troublesome/bothersome” the itch was (*n* = 2/15, 13.3% each). Adolescents understood the term “intensity” as “how itchy” or “itchiness” (*n* = 3/7, 42.9%), “how bad” (*n* = 2/7, 28.6%); and “severity,” “strength,” or “vigorousness” of itch (*n* = 1/7, 14.3%) (Table 2).

**Table 2:** Feedback on UAS Itch Item: itch intensity

UAS Itch Item	Participant feedback
**Itch Item: Please select the score that represents the intensity of your itch over the past 24 h.**	***100–105 Adult Female***: *I think for anybody who has CSU or anything that is itchy*,* we all understand the level of simply*,* moderate*,* bothering and to an intense bothering where it’s all you can think about….* ***100–108 Adult Female***: *It’s just asking me [about] the past 24 h…If I had gotten this question on a bad day*,* obviously my answers would be drastically different than on a good day.* ***100-002 Adolescent Male***: *It wasn’t difficult. It’s asking me like how bad I was itching or how the itching was affecting me throughout the 24 h.* ***00–006 Adolescent Male***: *How much they have itched and how often*,* I guess like have they been itching all the time…. Or how vigorous…has the itch been like*,* has been like an urging itch or just like a very light itch…From the beginning of today till the beginning of yesterday.*
**Response scale**: *Please choose one answer*: None Mild (present but not annoying or troublesome) Moderate (troublesome but does not interfere with normal daily activity or sleep) Intense (interferes with normal daily activity of sleep)	***100–107 Adult Female***: *The explanation next to the severity of—or the intensity [of itch 1] was helpful*,* like if it interferes with the everyday activity. I felt like in the parentheses what [was] explained—was helpful.* ***100–105 Adult Female***: *My answer to the first one is mild. Right now*,* it’s present but not annoying or troublesome. And each of the levels that they give you are very understandable*,* and I’ve been at each of those levels throughout my experience with CSU.* ***100–119 Adult Male***: *It was very easy to answer the question because when I looked at the moderate choice*,* it wasn’t troublesome enough*,* so I couldn’t give it a moderate because it wasn’t troublesome enough to cause me to lose my mind. So*,* I said moderate. I felt it*,* it was present and there were itchiness*,* but not troublesome. Not to the point where I couldn’t function*

*CSU* chronic spontaneous urticaria, *UAS* Urticaria Activity Score

***100-002 Adolescent Female***: It’s asking me like how bad I was itching or how the itching was affecting me throughout the 24 h.

***100-120 Adult Male***: It’s how I would identify my urticaria over the last 24 h … It says it plain English right here. Is it troublesome? Is it not very troublesome? Does it interfere with your daily life? And I would describe it as a nuisance. It’s annoying. It’s a distraction, but I can still get by with everyday life, including sleep. From this moment, until this exact same moment yesterday.

All participants (*n* = 22/22, 100.0%) liked the combined severity and impact response option descriptions of none, mild, moderate, and intense.

***100-105 Adult Female***: My answer to the first one is mild. Right now, it’s present but not annoying or troublesome. And each of the levels that they give you are very understandable, and I’ve been at each of those levels throughout my experience with CSU.

***100-107 Adult Female***: The explanation next to the severity of—or the intensity [for itch item] was helpful, like if it interferes with the everyday activity or, yeah, I felt like in the parentheses what explained—I felt like that was helpful.

Some participants (*n* = 6/22, 27.2%) provided suggestions for slightly adjusting the severity level descriptions to differentiate between intensity levels. For example, one adult participant felt that moderate intensity of the itch category should include interference with sleep or activity, especially since it is described as “troublesome.”

***100-104 Adult Female***: To be honest with you, moderate can still interfere with normal activity of sleep. So, it’s almost like you have to choose between not interfering with activity or sleep or interfering with activity or sleep, which means intense. So, I know the difference between moderate and intense. So presently with being moderate, because I know what my intense looks like, I do have trouble with activity or sleep, but it’s not intense, as far as I’m concerned. So, it’s making me choose based on what’s in the parentheses. So, I didn’t pay any attention to it. I just picked moderate, yeah. But it puts you in a spot where you have to choose, depending on the words, and I don’t think that’s really—I think what’s moderate and intense is individualized, regardless of the symptoms that goes with moderate or intense.

#### UAS itch intensity recall period

Most participants (adults *n* = 12/15, 80.0%; adolescents *n* = 6/7, 85.7%) understood assessing itch over 24 h as meaning “from the time of the interview to the same time, the day before.” All adolescent participants reported that it was “easy” or “not difficult” to recall their itch intensity over the past 24 h.

#### Meaningful change for itch item

**Fig. 1:**
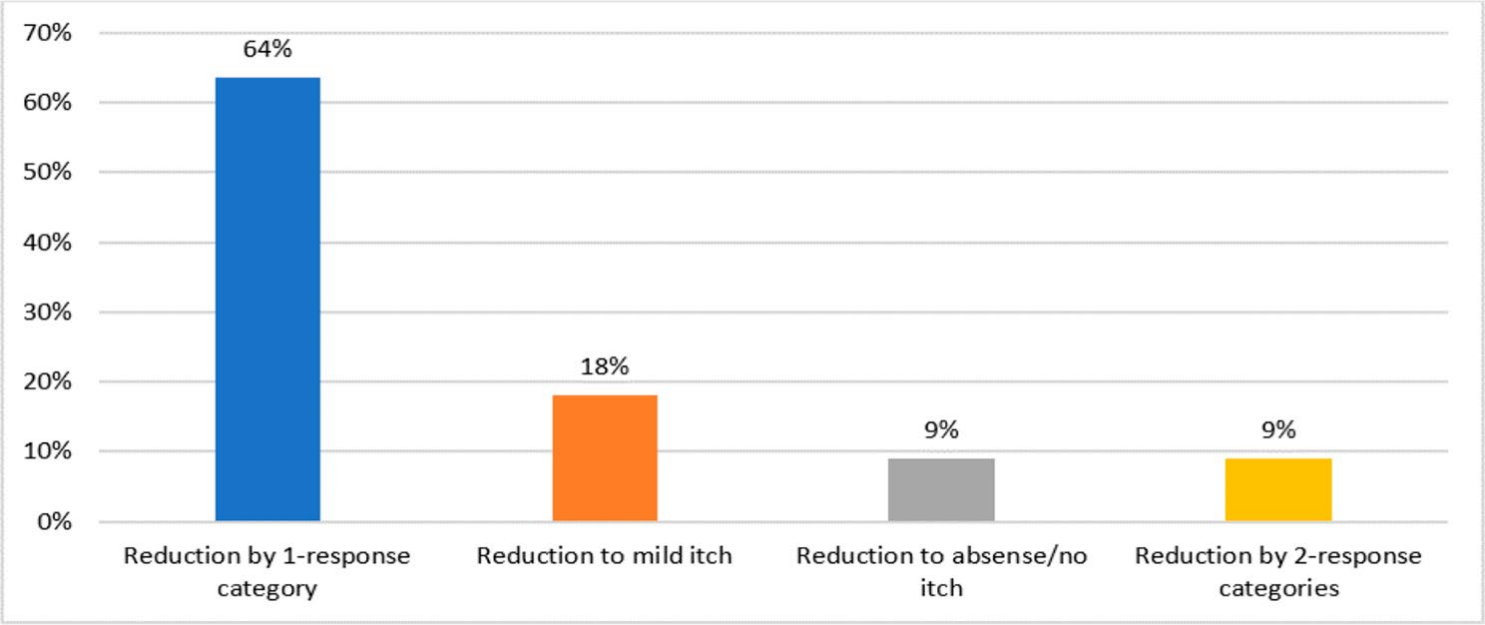
Meaningful change of itch severity

Most of the study sample (total, *n* = 14/22, 64.0%; adults, *n* = 8/15, 53.3%; adolescents, *n* = 6/7, 85.7%) confirmed that a one-category change would be a minimal meaningful improvement in itch intensity related to their CSU experience (Fig. 1). A minority of study participants (total, *n* = 4/22, 18.2%; adults *n* = 4/4, 100.0%) considered a move to mild itch or no itch from moderate or intense on the response scale to be a minimal meaningful improvement.

***100-102 Adult Female***: Probably going to just [be] mild every day, so like not having—I would expect to probably have hives, but not feeling itch, just eliminating the itch would probably be what I would consider helping…. Yeah, mild to no itching would probably be what I would expect.

***400-001 Adolescent Female***: I would probably say it should move to mild…. I think that I would feel like I can go outside more when it’s cold in the winter or go swimming without having to worry about it. But that doesn’t really affect my life a lot so it wouldn’t make too much of a change, but a good amount.

***100-001 Adolescent Male***: If the highest would go away a little shorter than they last usually, or if they don’t itch as much as they used to: Probably close to none.

#### Understanding of UAS wheals (hives) item

All participants (*n* = 22/22, 100.0%) were able to describe the UAS wheals (hives) items in their own words and demonstrated clear understanding of the item. Most adults (*n* = 14/15, 93.3%) understood wheals as “hives,” since “hives” was provided in parentheses. All adolescents understood “wheals” to mean “hives.” Only four participants (total, *n* = 4/22, 18.2%; adults, *n* = 2/15, 13.3%; adolescents *n* = 2/7, 28.6%) were familiar with the word “wheals” before the interview. Most adults and adolescents (*n* = 20/22, 90.9%) understood the term “score that corresponds” to mean “pick the number that best describes or corresponds,” “the best estimate of the number of hives,” “choose the number that matches,” “count of individual bumps,” “ranking,” etc. (Table 3).

**Table 3:** Feedback on UAS wheals (hives) item: count of wheals/hives

UAS Wheals (hives) Item	participant feedback
**Wheals (hives) Item: Please select the score that corresponds to the number of wheals you have had over the past 24 h.** **For a group of overlapping hives**,** count separately as best as possible**	***100–105 Adult Female***: *Had it just said wheals*,* I would have thought about it*,* and I had looked it up*,* but using the words together help anybody understand that these are the bumps that you have that are irritated*,* raised. So that was easy to understand.* ***200-006 Adolescent Male***: *I didn’t know they were called wheals*,* but I just called them hives*,* so it just made sense*,* like there didn’t need to be any added explanation. It just had the hives in parenthesis*,* so I understood.* ***100–108 Adult Female***: *“[UAS Wheals (hives) item] it’s asking me to count the number of welts. For my flares*,* like the welts are actually quite large. They’re like large patches of raised skin. So*,* it’s not like I’m covered in a bunch of like tiny hives. It’s more like large masses of skin*,* so easy to count. And I think the way that this was broken up*,* like the quantity*,* is like pretty reasonable.”* ***100–116 Adult Male***: *No*,* I think this was unclear*,* and I think it was clear from my understanding. It was clear and easy to understand.*
**Response scale**: None <20 20–50 >50	***400-001 Adolescent Female***: *I had a hard time choosing between 20 to 50 and greater than 50 just because I know it was—I know it was more than 20*,* but it’s like a big range for me to be able to decipher if it was more or less than 50.* ***100–112 Adult Female***: *As someone who works in the education field*,* I feel that some people might get tripped up by the greater than/less than signs. That might be difficult for some people … I think if it just said less than or more than in written words*,* it might be more clear across the board for everyone.* ***200-006 Adolescent Male***: *Well*,* at the moment*,* whenever I saw the … the greater than 20*,* I had to think back to math. The greater than or less than. Well*,* I just had to think a little…* ***100–116 Adult Male***: *I feel like maybe the only tricky bit about this is the greater and the lesser than sign and then the numbers*,* just that. I think the mathematical figures just make it a little bit more—it made it kind of feel more medical … like something that only those working in the medical field … would really…understand.*

***200-006 Adolescent Male***: I didn’t know they were called wheals, but I just called them hives, so it just made sense, like there didn’t need to be any added explanation. It just had the hives in parenthesis, so I understood.

***100-105 Adult Female***: Had it just said wheals, I would have thought about it, and I had looked it up, but using the words together help anybody understand that these are the bumps that you have that are irritated, raised. So that was easy to understand.

#### UAS wheals (hives) item recall period

Participants were asked how easy it was for them to think back and count hives in the past 24 h. Most participants (total, *n* = 15/22, 68.2%; adults, *n* = 9/15, 60.0%; adolescents, *n* = 6/7, 85.7%) confirmed that hives over the past 24 h meant from “the time of the interview to the same time the day before.” Most adults (*n* = 10/15, 66.7%) and adolescents (*n* = 4/7, 57.1%) reported that it was “easy” or “not difficult” to count and recall their number of hives over the past 24 h. Based on participant feedback, instructions were added to use a mirror or photograph to view hives in sensitive areas.

Some participants noted that counting hives over a 24-hour period was difficult. However, they were easily able to select an answer from the response categories because they included a range of the number of hives in the past 24 h. Almost half of the adults (*n* = 7/15, 47.0%) and less than one-third of the adolescents (*n* = 2/7, 29.0%) experienced < 20 hives in the past 24 h.

A few participants (total, *n* = 4/22, 18.2%; adults *n* = 3/15, 20.0%; adolescents *n* = 1/7, 14.3%) also mentioned that they thought the 20-to-50 response option was quite a wide range. A small proportion of adults (*n* = 2/15, 13.3%) but a larger proportion of adolescents (*n* = 3/7, 42.9%) were slightly confused with the mathematical symbols (>, < ) used in the response scale.

***200-006 Adolescent Male***: Well, at the moment, whenever I saw the … the greater than 20, I had to think back to math. The greater than or less than. Well, I just had to think a little….

***100-116 Adult Male***: I feel like maybe the only tricky bit about this is the greater and the lesser than sign and then the numbers, just that. I think the mathematical figures just make it a little bit more—it made it kind of feel more medical, more professional. Like just it made it feel like something that only those working in the medical field or professional field would really get to understand.

***100-112 Adult Female***: I will say as someone who works in the education field, I feel that some people might get tripped up by the greater than/less than signs. That might be difficult for some people. But for me, it wasn’t a problem. But I think if it just said less than or more than in written words, it might be more clear across the board for everyone.

#### Meaningful change for wheals (hives) item

**Fig. 2:**
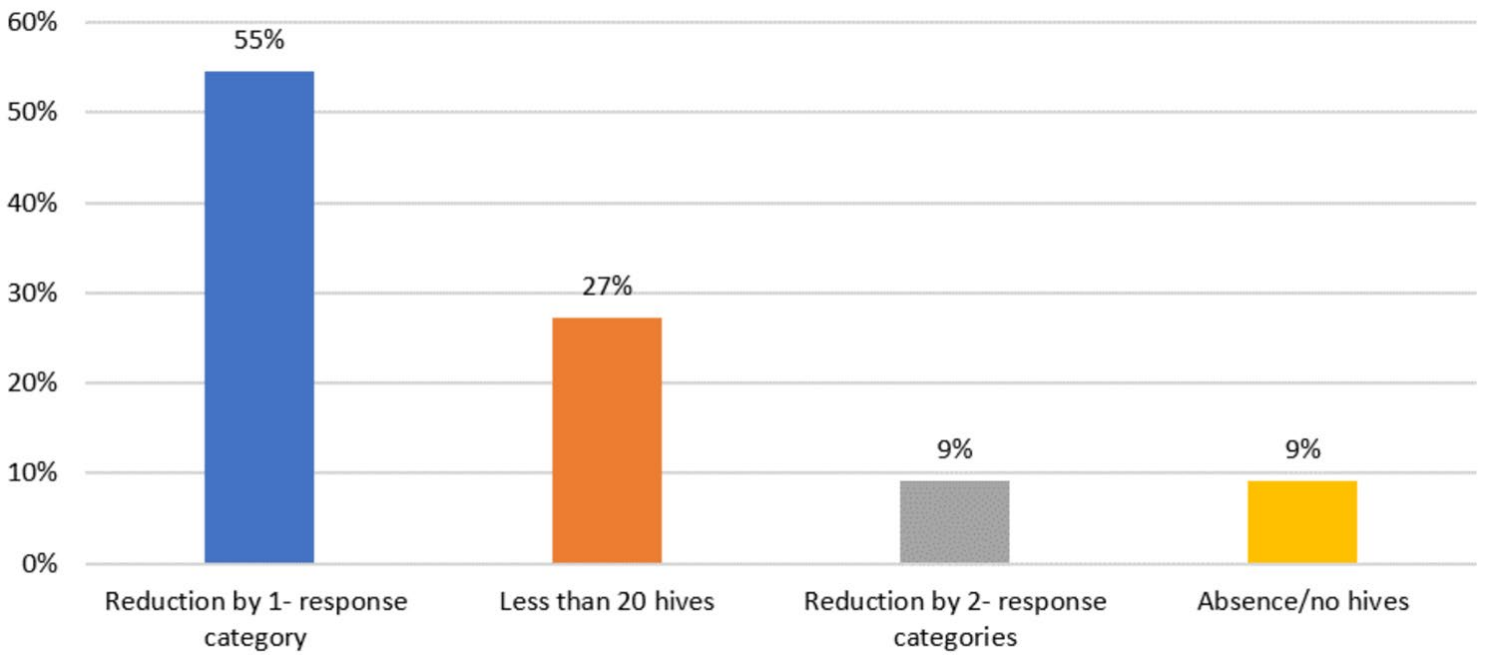
Meaningful change–count of wheals (hives)

When participants were asked broadly what level of change would indicate that the treatment was reducing their number of hives, participants described improvements as “consistent reductions” in the number of hives, as well as reductions in itch frequency, itch intensity, and interference with daily activities. When asked specifically what level of change on the UAS wheals (hives) item would be minimally meaningful, most of the study sample (total, *n* = 12/22, 54.5%; adults, *n* = 8/15, 53.3%; adolescents, *n* = 4/7, 57.1%) responded that a one-category change would be meaningful. One-third of adolescents (28.6%) reported that a two-category change in the score for number of hives would be necessary to be considered meaningful (Fig. 2).

***100-002 Adolescent Female***: It would have to be like less than 15 [from 20 to 50] on a normal day with like PE [physical education] or volleyball, basketball, all that. It’s going to be very meaningful. I would be very happy knowing I would have less on my body. I would be very happy about that.

***100-116 Adult Male***: I think if I should move from 50 to 20 or to none. None would be the perfect change or the final results that I would expect. But being realistic, most of these things don’t just disappear like that. They take time to disappear, so I agree we should just reduce gradually from 50 to 20 and then to none.

***400-003 Adolescent Male***: If on average my hives decreased [from 20 to 50 to] Probably less than 20. It would decrease the stress and intensity of, like, itchiness, and I could concentrate more on, like, schoolwork.

#### Feedback on UAS patient guidance document

Participants found the guidance document “helpful” and “easy to understand.” They especially appreciated the instructions for determining and counting hives over a 24-hour recall period.

***100-001 Adolescent Male***: It [patient guidance document] was helpful. It was pretty helpful.

***100-112 Adult Female***: Yes … So having very clear descriptions is comforting.

***100-103 Adult Female***: It [patient guidance document] was very helpful. It was to the point, and it read well in layman’s terms.

***300-007 Adolescent Male***: It [patient guidance document] was moderately helpful. I figured I just read the question and answered based on the question.

The patient guidance document helped participants understand the terms “score,” “wheals,” and “overlapping hives” in relation to counting their hives. Only minor suggestions were made for clarifying sections of the guidance document, most notably adding a photograph to help with counting hives (*n* = 3/15, 20%).

***100-105 Adult Female***: The second question asks you about the number of wheals or hives you’ve had in the past 24 h … And I don’t know if a diagram or a picture … of some part, a leg or an arm, that shows a bunch [of hives] … would just give them some visual help.

***100-119 Adult Male***: Bumps, people would understand, but maybe you need to have a diagram in there, a diagram, a picture of forming a patch of more—of hives forming a patch. I think maybe if you have an illustration in there, even if you have an illustration of bumps, this would help people count them, and this would help people who are counting the ones on your back to count them correctly.

## Discussion

The UAS is the gold standard for measuring itch and hives and is a valid tool for measuring and monitoring disease activity in patients with CSU [10]. The purpose of this study was to assess the relevance and comprehension of the modification made to the item 2 to add “(hives)” to the US English version and the usefulness of the patient guidance document for use in adult and adolescent populations with CSU.

The concept elicitation portion of the interviews confirmed that itch and hives were relevant and important to patients’ CSU sign and symptom experience. The key cognitive interview findings indicated that adult and adolescent participants had positive feedback and found the adapted UAS to be relevant, straightforward to complete, and easy to understand—especially when provided with the additional instruction from the UAS patient guidance document.

Participants had a clear understanding of the adapted UAS items, recall period, and response options. Study participants reported that they used a 24-hour recall period when responding to the adapted UAS. When asked to describe the items in their own words, all participants were able to do so, and demonstrated clear understanding. Most adults and adolescents agreed that a one-category change on the itch and hives UAS items would be a meaningful improvement. Participants’ perception of what constitutes a minimal meaningful change on each UAS item can aid in interpreting minimal meaningful change on the UAS7 score. This qualitative data provides preliminary patient insight experiences into meaningful change on the UAS items. Knowing how UAS7 scores relate to patients’ experiences is valuable to interpreting the meaningfulness of the UAS7 endpoint.

The guidance document was deemed important to help patients complete the adapted UAS questionnaire and maximize the comprehensibility of the instrument across individuals with varying health literacy levels. Based on participant suggestion, a photograph of hives was incorporated into the guidance document to assist in identifying and counting hives.

### Limitations

The sample of adults and adolescents was not representative of individuals with CSU in the US and may not completely reflect the views of all members of this diverse group. This is a limitation inherent to all studies that use convenience sampling methods. To overcome this limitation, diversity was sought in age, race/ethnicity, health literacy, and geographic location of adult and adolescent samples. The final sample (*n* = 22) included fewer than the planned number of participants (*n* = 30). However, saturation results demonstrated that concept saturation was reached, indicating a sufficient sample size for content validation. These qualitative findings provide preliminary evidence of a one-category change for the UAS items as a minimally meaningful change as a starting point for further validation steps of the adapted UAS.

## Conclusions

Itch and hives are relevant and important symptoms and signs—central to patient experiences of CSU. The adapted UAS demonstrated content validity as a daily PRO measure of severity of itch and hives for adolescents ≥ 12 years old and adults with CSU. The qualitative interviews provided valuable suggestions for improving its patient guidance, which can be used in future clinical trials involving adults and adolescents with CSU to assess the severity of itch and hives.

## Electronic supplementary material

Below is the link to the electronic supplementary material.

Supplementary Material 1

## Data Availability

All data generated or analysed during this study are included in this published article [and its supplementary information files].

## Abbreviations

CSU: Chronic spontaneous urticaria
ISPOR: Professional Society for Health Economics and Outcomes Research
PRO: Patient-reported outcome
UAS: Urticaria Activity Score
UAS7: 7-day Urticaria Activity Score
