# Supplementary material for: Comprehension of the adapted Urticaria Activity Score measure and patient guidance document: qualitative interviews with adults and adolescents with chronic spontaneous urticaria
Source: J Patient Rep Outcomes. 2024 Dec 24;8:153. doi: 10.1186/s41687-024-00830-9 (PMC11668714; doi:10.1186/s41687-024-00830-9)
Supplement: Supplementary file 1 — Supplementary Material 1 [file 41687_2024_830_MOESM1_ESM.docx]

# Supplementary Material

Supplemental Table 1. Detailed Demographic and Clinical Characteristics

| Demographic Characteristic | Total (N=22) | Adolescents (n=7) | Adults (n=15) |
| --- | --- | --- | --- |
| Age (years) |  |  |  |
| N | 22 | 7 | 15 |
| Mean (SD) | 34.0 (17.9) | 14.3 (1.8) | 43.3 (13.8) |
| Median (range) | 33.5 (12–65) | 14.0 (12–17) | 40.0 (23–65) |
| Sex, n (%) |  |  |  |
| Male | 8 (36.4%) | 4 (57.1%) | 4 (26.7%) |
| Female | 14 (63.6%) | 3 (42.9%) | 11 (73.3%) |
| Employment Status, n (%) |  |  |  |
| Employed, full time | 10 (45.5%) | 0 (0.0%) | 10 (66.7%) |
| Student | 9 (40.9%) | 7 (100.0%) | 2 (13.3%) |
| Retired | 1 (4.5%) | 0 (0.0%) | 1 (6.7%) |
| Disabled | 2 (9.1%) | 0 (0.0%) | 2 (13.3%) |
| Highest education level, n (%) |  |  |  |
| Some high school, but no diploma | 3 (13.6%) | 3 (42.9%) | 0 (0.0%) |
| University/college degree | 10 (45.5%) | 0 (0.0%) | 10 (66.7%) |
| Professional or advanced degree (i.e., MBA, PhD) | 5 (22.7%) | 0 (0.0%) | 5 (33.3%) |
| Other^a^ | 4 (18.2%) | 4 (57.1%) | 0 (0.0%) |
| Ethnic background, n (%) |  |  |  |
| Hispanic or Latino | 5 (22.7%) | 1 (14.3%) | 4 (26.7%) |
| Not Hispanic or Latino | 17 (77.3%) | 6 (85.7%) | 11 (73.3%) |
| Racial background, n (%) |  |  |  |
| Asian | 1 (4.5%) | 0 (0.0%) | 1 (6.7%) |
| Black or African American | 3 (13.6%) | 2 (28.6%) | 1 (6.7%) |
| White | 17 (77.3%) | 5 (71.4%) | 12 (80.0%) |
| Other^b^ | 1 (4.5%) | 0 (0.0%) | 1 (6.7%) |
| Current relationship status, n (%) |  |  |  |
| Married/living as married/partnered | 8 (36.4%) | 0 (0.0%) | 8 (53.3%) |
| Single | 14 (63.6%) | 7 (100.0%) | 7 (46.7%) |
| Geographic region of residence, n (%) |  |  |  |
| Northeast | 4 (18.2%) | 0 (0.0%) | 4 (26.7%) |
| Southeast | 7 (31.8%) | 2 (28.6%) | 5 (33.3%) |
| Midwest | 7 (31.8%) | 2 (28.6%) | 5 (33.3%) |
| Southwest | 3 (13.6%) | 3 (42.9%) | 0 (0.0%) |
| Pacific | 1 (4.5%) | 0 (0.0%) | 1 (6.7%) |
| Health literacy/confidence |  |  |  |
| Extremely confident | 14 (63.6%) | 1 (14.3%) | 13 (86.7%) |
| Quite confident | 4 (18.2%) | 2 (28.6%) | 2 (13.3%) |
| Fairly confident | 3 (13.6%) | 3 (42.9%) | 0 (0.0%) |
| A little confident | 0 (0.0%) | 0 (0.0%) | 0 (0.0%) |
| Not confident | 1 (4.5%) | 1 (14.3%) | 0 (0.0%) |
| **Clinical Characteristics** | | | |
| Number of years since diagnosed (years) |  |  |  |
| Mean (SD) | 4 (3.2) | 4 (3.2) | 2 (2.3) |
| Time on current treatment (years) |  |  |  |
| Mean (SD) | 2.7 (2.3) | 2.4 (2.6) | 2.8 (2.1) |
| Current treatment–method of delivery,  n (%) |  |  |  |
| Pill | 54 (76.1%) | 19 (82.6%) | 35 (72.9%) |
| Injection | 10 (14.1%) | 1 (4.3%) | 9 (18.8%) |
| Cream | 7 (9.9%) | 3 (13.0%) | 4 (8.3%) |
| Reason for discontinuing prior medication, n (%) |  |  |  |
| Did not see need | 2 (2.8%) | 0 (0.0%) | 2 (4.2%) |
| Other reason^c^ | 4 (5.6%) | 0 (0.0%) | 4 (8.3%) |
| General health, n (%) |  |  |  |
| Excellent | 1 (4.5%) | 1 (14.3%) | 0 (0.0%) |
| Very good | 9 (40.9%) | 5 (71.4%) | 4 (26.7%) |
| Good | 8 (36.4%) | 1 (14.3%) | 7 (46.7%) |
| Fair | 4 (18.2%) | 0 (0.0%) | 4 (26.7%) |
| Poor | 0 (0%) | 0 (0%) | 0 (0%) |
| UAS Itch Item |  |  |  |
| Mean (SD) | 2.6 (1.0) | 2.5 (0.9) | 2.7 (1.1) |
| None | 3 (13.6%) | 2 (13.3%) | 1 (14.3%) |
| Mild (present but not annoying or troublesome) | 7 (31.8%) | 5 (33.3%) | 2 (28.6%) |
| Moderate (troublesome but does not interfere with normal daily activity or sleep) | 8 (36.3%) | 6 (40.0%) | 2 (28.6%) |
| Intense (interferes with normal activity or sleep) | 4 (18.2%) | 2 (13.3%) | 2 (28.6%) |
| UAS Wheals (hives) Item |  |  |  |
| Mean (SD) | 2.4 (0.9) | 2.7 (1.1) | 2.1 (0.7) |
| None | 4 (18.1%) | 1 (14.3%) | 3 (20.0%) |
| <20 | 9 (40.9%) | 2 (28.6%) | 7 (46.7%) |
| 20 to 50 | 7 (31.8%) | 2 (28.6%) | 5 (33.3%) |
| >50 | 2 (9.1%) | 2 (28.6%) | 0 (0%) |

*SD* standard deviation**,** *UAS* Urticaria Activity Score

^a^ Other education: 8th grade/junior high school.

^b^ Other race: Jewish.

^c^ Other reason: Doctor-recommended, hair started falling out and was experiencing fatigue, no relief.

# Supplementary Material 2: Patient Guidance Document for Completing the UAS Questionnaire

**Patient Guidance for Completing the Urticaria Activity Score (UAS)**

This patient guidance explains how you should complete the Urticaria Activity Score (UAS) questionnaire. Please review this guidance to help complete the UAS each day in your electronic handheld device.

## What is the UAS and why am I completing it?

The UAS is a questionnaire that asks about the severity of your chronic spontaneous urticaria (CSU).

The UAS includes two questions:

- The first question asks you to rate the intensity or severity of your itch in the past 24 hours.
- The second question asks you about the number of wheals (or hives) you had in the past 24 hours.

Please answer these questions each day, around same time in the evening on the electronic device.

Your answers to these questions during the clinical study will help us understand changes in the severity of your CSU and see if the study treatment has helped you.


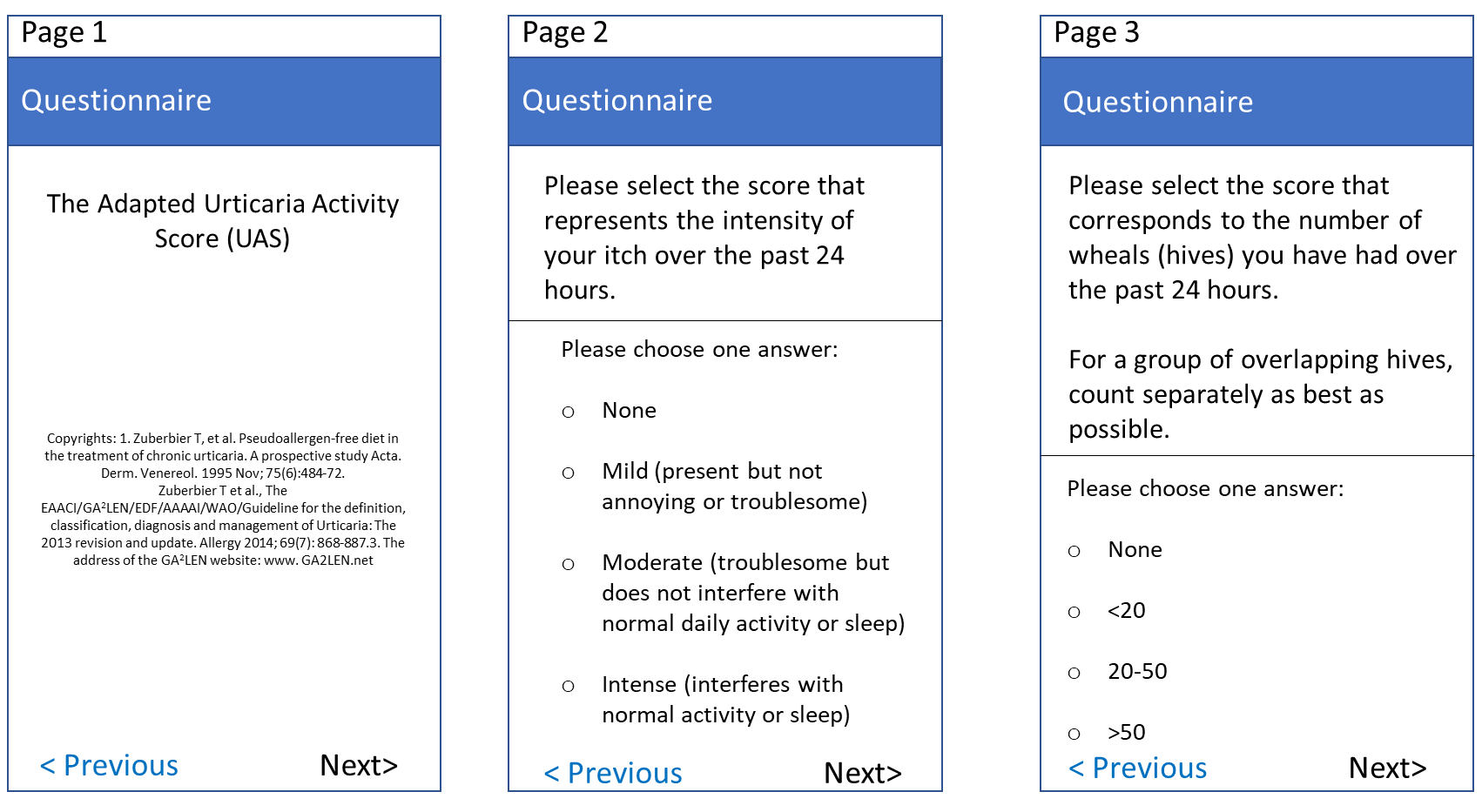


## When and how do I answer the questions on the UAS?

## How do I rate the intensity / severity of itch and number of wheals (hives) on your body?

When completing the UAS, think back over the last 24 hours and how severe was your itch or the number of wheals (hives) on your body.

Please complete the UAS questions around the same time every evening.

*How do I answer the first question- Intensity of Itch?*

For this question, you should choose the one answer that best describes the intensity (severity) of your itch at its worst over the past 24 hours.

*How do I answer the second question–Number of Wheals (Hives)?*

For this question, you should choose the one answer that best describes the number of wheals (hives) you have had on your body over the past 24 hours.

The box below gives you additional information to help you count the number of wheals (hives).

| **What are wheals (hives)?** | **How do I count the number of wheals (hives)?** |
| --- | --- |
| Wheals (hives) can look like:   - Raised bumps that are red, pink, or skin-colored - Raised smooth areas of the skin that can vary in size - Can be itchy and can sometimes burn or sting - Can last minutes, hours, days or much longer - Wheals (hives) can join together to form a large size patch | Please count the total number of wheals (hives) on your body over the past 24 hours.  For example, if yesterday around noon   - - you had **10** wheals (hives) on your left arm, but they were gone after 30 minutes   And later in the afternoon   - - you had **15** wheals (hives) in the same arm and **5** wheals (hives) on your right arm   In this example, count up the total number of wheals (hives) 10 + 15 + 5 = 30 in the past 24 hours |
| IMPORTANT | |
| If two or more wheals (hives) join together or form a patch, count each one separately. | |
| If wheals (hives) appear in areas of your body that you can’t see by yourself,  for example, on your back, you can ask someone to help you with the counting.  If you do ask someone to help you, please have them read this guide  to make sure that they count your wheals (hives) correctly | |

If you have any questions or are not sure how to assess your itch and/or wheals (hives), please talk to your study doctor.

# Supplementary Material 3: Adapted UAS


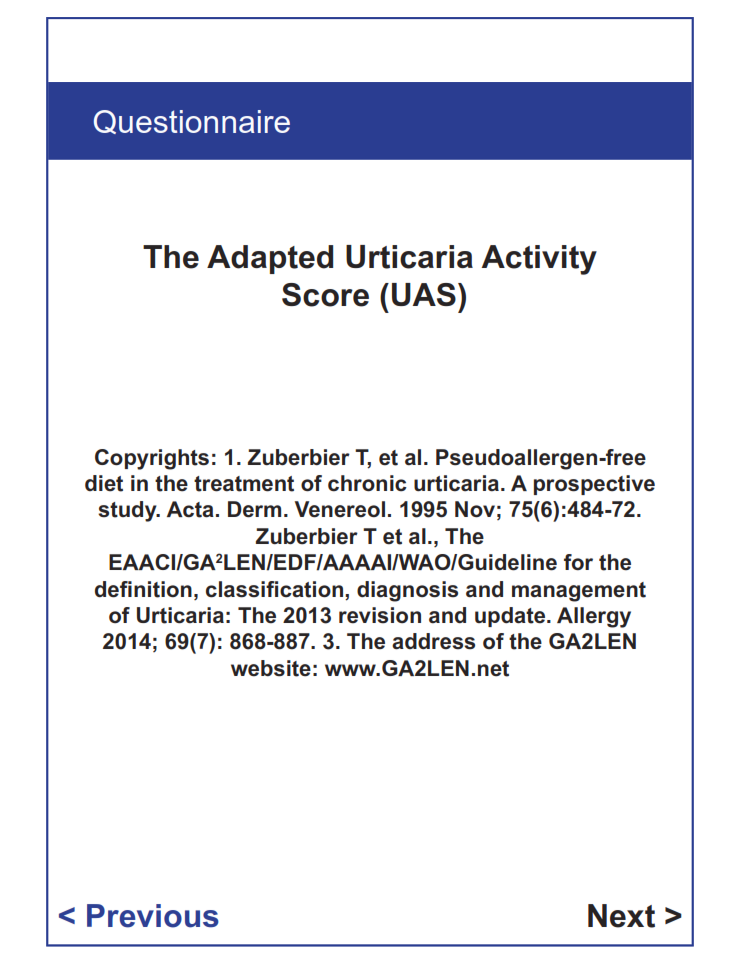

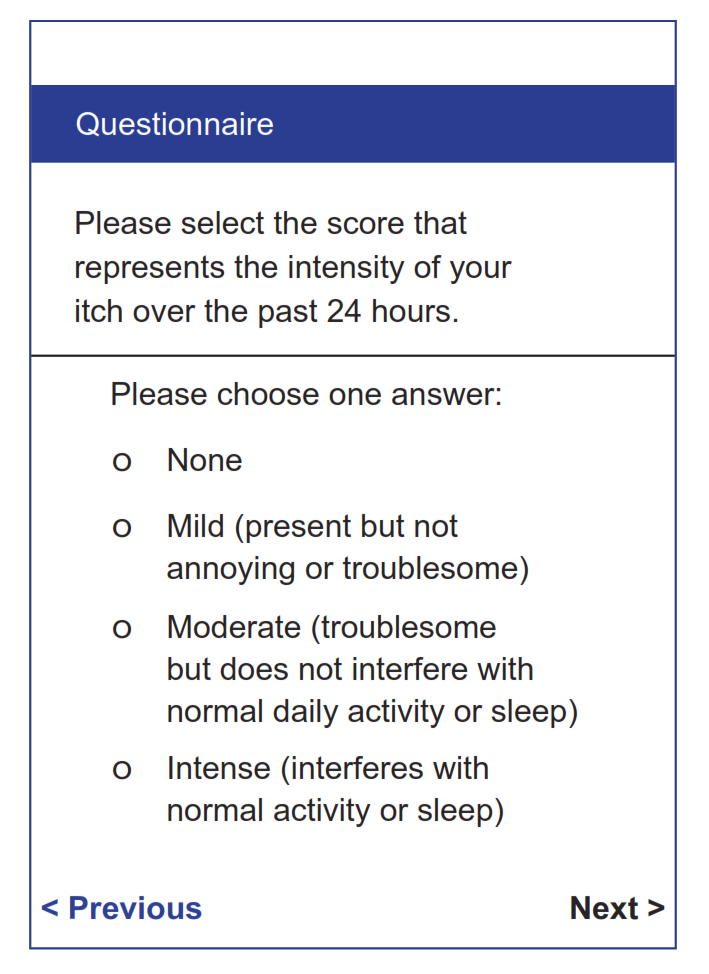

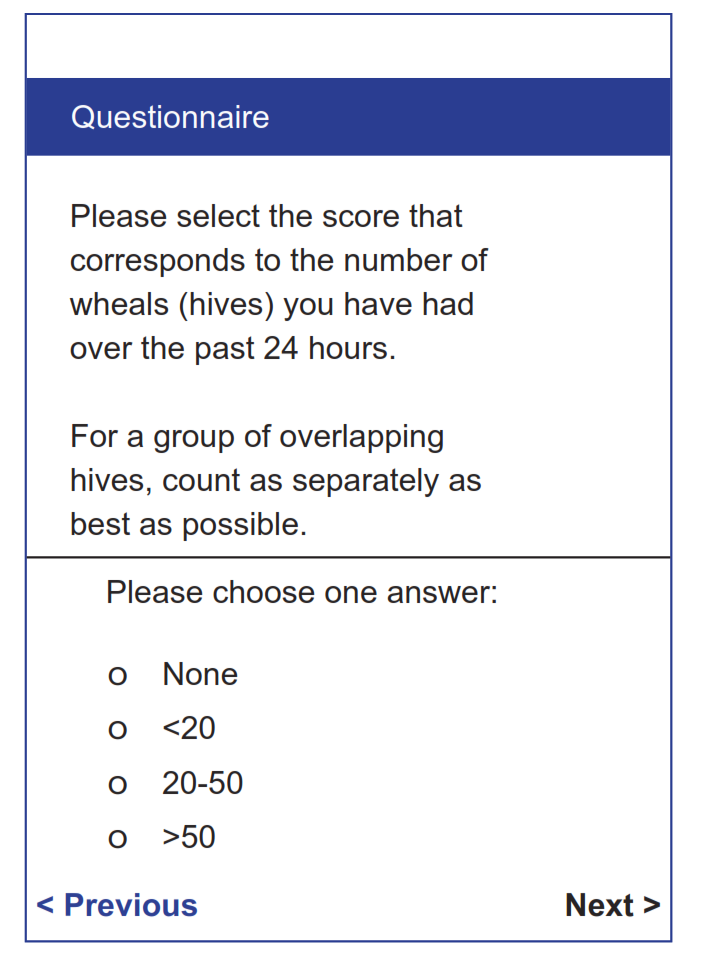
3
